# Theta–gamma coupling as a cortical biomarker of brain–computer interface-mediated motor recovery in chronic stroke

**DOI:** 10.1093/braincomms/fcac136

**Published:** 2022-05-25

**Authors:** Nabi Rustamov, Joseph Humphries, Alexandre Carter, Eric C. Leuthardt

**Affiliations:** Department of Neurological Surgery, Washington University School of Medicine, St Louis, MO, USA; Center for Innovation in Neuroscience and Technology, Washington University School of Medicine, St Louis, MO, USA; Department of Biomedical Engineering, Washington University in St Louis, St Louis, MO, USA; Department of Neurology, Washington University in St Louis, St Louis, MO, USA; Department of Neurological Surgery, Washington University School of Medicine, St Louis, MO, USA; Center for Innovation in Neuroscience and Technology, Washington University School of Medicine, St Louis, MO, USA; Department of Biomedical Engineering, Washington University in St Louis, St Louis, MO, USA; Department of Neuroscience, Washington University School of Medicine, St Louis, MO, USA; Department of Mechanical Engineering and Materials Science, Washington University in St Louis, St Louis, MO, USA

**Keywords:** chronic stroke rehabilitation, brain–computer interface, theta–gamma coupling

## Abstract

Chronic stroke patients with upper-limb motor disabilities are now beginning to see treatment options that were not previously available. To date, the two options recently approved by the United States Food and Drug Administration include vagus nerve stimulation and brain–computer interface therapy. While the mechanisms for vagus nerve stimulation have been well defined, the mechanisms underlying brain–computer interface-driven motor rehabilitation are largely unknown. Given that cross-frequency coupling has been associated with a wide variety of higher-order functions involved in learning and memory, we hypothesized this rhythm-specific mechanism would correlate with the functional improvements effected by a brain–computer interface. This study investigated whether the motor improvements in chronic stroke patients induced with a brain–computer interface therapy are associated with alterations in phase–amplitude coupling, a type of cross-frequency coupling. Seventeen chronic hemiparetic stroke patients used a robotic hand orthosis controlled with contralesional motor cortical signals measured with EEG. Patients regularly performed a therapeutic brain–computer interface task for 12 weeks. Resting-state EEG recordings and motor function data were acquired before initiating brain–computer interface therapy and once every 4 weeks after the therapy. Changes in phase–amplitude coupling values were assessed and correlated with motor function improvements. To establish whether coupling between two different frequency bands was more functionally important than either of those rhythms alone, we calculated power spectra as well. We found that theta–gamma coupling was enhanced bilaterally at the motor areas and showed significant correlations across brain–computer interface therapy sessions. Importantly, an increase in theta–gamma coupling positively correlated with motor recovery over the course of rehabilitation. The sources of theta–gamma coupling increase following brain–computer interface therapy were mostly located in the hand regions of the primary motor cortex on the left and right cerebral hemispheres. Beta–gamma coupling decreased bilaterally at the frontal areas following the therapy, but these effects did not correlate with motor recovery. Alpha–gamma coupling was not altered by brain–computer interface therapy. Power spectra did not change significantly over the course of the brain–computer interface therapy. The significant functional improvement in chronic stroke patients induced by brain–computer interface therapy was strongly correlated with increased theta–gamma coupling in bihemispheric motor regions. These findings support the notion that specific cross-frequency coupling dynamics in the brain likely play a mechanistic role in mediating motor recovery in the chronic phase of stroke recovery.

## Teaser

Rustamov *et al*. report that theta–gamma coupling enhanced bilaterally at the motor regions following brain–computer interface therapy showing a positive correlation with motor recovery. These findings imply that specific cortical cross-frequency coupling dynamics likely play a mechanistic role in mediating motor recovery in chronic stroke.

## Graphical Abstract

**Graphical abstract:**
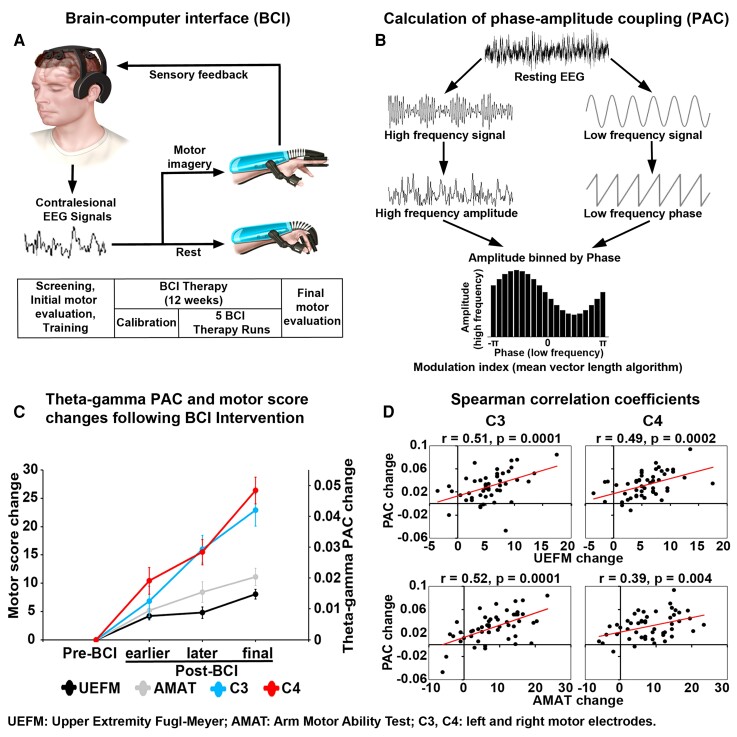

## Introduction

About two-thirds of stroke patients suffering from hemiparesis are still unable to fully use their affected limb 6 months after stroke.^1–3^ Motor recovery usually plateaus at 3 months post-stroke, and residual motor deficits ultimately become permanent.^4–8^ Trials of increased rehabilitation therapy dose or brain stimulation therapies have not been effective.^9–11^ Developing new treatments for stroke rehabilitation remains a research priority. Vagus nerve stimulation therapy combined with movement training has been shown to help achieve improvement in upper-limb motor recovery in patients with chronic stroke,^12–14^ possibly through cholinergic and monoaminergic modulation of motor cortex neurons.^15,16^ Moreover, studies using neuroprosthetic strategies for stroke rehabilitation have shown that functional improvements can be achieved even in the chronic stage.^17–21^ One approach is the application of a brain–computer interface (BCI)-controlled robotic hand orthosis using EEG signals from the contralesional motor cortex.^22–24^ Contralesionally controlled BCI therapy has been shown to facilitate motor rehabilitation in severely impaired chronic stroke patients.^23^ However, the mechanisms underlying BCI-driven motor rehabilitation are poorly understood. Defining changes in cortical electrophysiology with motor recovery in the chronic phase of stroke will better elucidate the mechanisms promoting motor learning and facilitate further refinement of motor rehabilitation strategies.

Previous studies have supported the role of the contralesional hemisphere in post-stroke recovery. Functional MRI (fMRI) studies of stroke patients have shown that increased contralesional activity is associated with improved motor function.^25,26^ The use of the uninjured motor cortex as the control signal for BCI rehabilitation further demonstrated the beneficial role of the unaffected hemisphere in motor recovery.^23^ Conversely, several studies have shown that the reduction of the contralesional motor cortical activity enhances motor function in the affected limb of hemiparetic stroke patients, which suggests that the contralesional hemisphere impedes recovery.^27–30^ Taken together, there is increasing support that the unaffected motor cortex plays a role in motor recovery, but underlying physiological mechanisms require further clarification. In previous work in animal models, there has been substantial evidence that M1 plays a role in the acquisition of motor skills.^31–33^ In humans, the cortical physiology associated with motor learning in M1 is more limited.^34,35^ This physiology in the setting of chronic stroke is even more scarce (see Kantak *et al*.^36^ for a review).

Coupling between different frequency bands may be a potential mechanism for motor learning. Traditionally, neural oscillations have been divided into specific frequency bands and studied according to their spectral features alone.^37,38^ Higher-frequency oscillations (>70 Hz), known as gamma rhythms, are thought to represent local cortical ensembles.^39,40^ Narrow bands under 30 Hz, such as theta (4–7 Hz), alpha (8–12 Hz) and beta rhythms (13–29 Hz), have been posited to represent modulatory circuits associated with deeper grey structures such as the thalamus and hippocampus.^41–43^ In the recent years, there is growing interest in exploring more complex properties of neural oscillations, such as synchronization between the phase of low-frequency oscillations and the amplitude of higher-frequency oscillations, i.e. phase–amplitude coupling (PAC), a type of cross-frequency coupling (CFC).^44–48^ It has been suggested that PAC reflects the regulation of high-frequency local oscillation by a larger network oscillating at lower frequencies.^49^ PAC has been associated with a wide variety of higher-order functions involved in learning and memory,^50–54^ attention,^55,56^ nociception,^57,58^ motor and visuomotor tasks.^59–64^ The mechanisms underlying learning have been most extensively studied in the hippocampus, where theta–gamma PAC has been hypothesized as a key learning-related mechanism.^46,54,65–67^ It has been determined that theta–gamma PAC also plays a similar role in learning throughout the neocortical regions.^49,68^ As in the hippocampus, M1 gamma oscillations are modulated by theta activity through PAC.^69^ In a preliminary study, enhancement of theta–gamma PAC via transcranial alternating current stimulation (tACS) over M1 during learning of motor skills resulted in a significant improvement in motor skill acquisition.^70^ This implies a potential role of theta–gamma PAC in motor skill learning but requires further investigation.

In this study, we sought to evaluate in chronic stroke patients whether BCI therapy-induced motor improvement is associated with alterations in PAC between gamma and lower frequencies. Contralesionally controlled BCI training used cortical signals related to affected hand motor imagery, recorded from the unaffected hemisphere, to control the affected hand via a powered hand exoskeleton. Resting-state EEG recordings of patients with chronic stroke were examined throughout a 12-week period of BCI training. Given the prior evidence showing the potential implications of theta–gamma PAC in motor learning, we hypothesized that theta–gamma PAC will be primarily changed with BCI intervention and that these changes over motor areas will correlate with the magnitude of motor recovery. As in prior studies, chronic stroke patients achieved a clinically significant motor recovery following BCI therapy.^22–24^ Here, we found a significant increase in theta–gamma PAC over motor areas which positively correlated with these functional improvements. These findings highlight an important role of theta–gamma PAC enhancement in the facilitation of motor improvement which may represent a key underlying mechanism for motor learning with the use of a BCI therapy in chronic stroke patients.

## Materials and methods

### Study population

Seventeen chronic stroke patients with upper-limb hemiparesis completed the full course of BCI therapy for 12 weeks. The inclusion criteria were the following: stroke at least 6 months prior confirmed by neurologist or medical records; intact cognitive ability quantified by a score of 0–1 on Items 1b and 1c (cognition) of the NIH Stroke Scale; unilateral upper extremity weakness; ability to provide informed consent; full passive range of motion of the affected elbow, wrist and digits and normal sensation (tactile and proprioceptive) in the affected upper extremity. The exclusion criteria were the following: severe visual impairment; cognitive impairment (8 or more on the Short Blessed Test); Botox injections in the affected upper extremity for spasticity management in the prior 3 months; severe aphasia, ataxia or unilateral neglect; severe psychiatric disorders such as schizophrenia or pre-stroke bipolar disorder; concurrent participation in other stroke studies. All patients suffered a first-time stroke at least 6 months prior to this study. Patient demographics are shown in Table 1 (see the ‘Results’ section). Motor function outcomes were primarily assessed with the upper extremity Fugl-Meyer (UEFM) assessment, which has been validated in a stroke patient population and has high reliability.^71–73^ Secondary motor function outcomes were measured using the Arm Motor Ability Test (AMAT), motricity index (MI), modified Ashworth Scale (MAS) at the wrist and elbow and grip strength. This study was approved by the Institutional Review Board of Washington University School of Medicine in St Louis. The data in this study were pooled across two pre-registered studies (NCT04338971 and NCT03611855) with identical research protocols. Before data collection, all patients gave written informed consent according to the Declaration of Helsinki.

**Table 1:** Patient demographics and primary motor assessment scores (mean ± SEM)

Age (years)	Time since stroke (months)	BCI usage (h)	Lesion side: hemisphere	Gender	Baseline UEFM	Final UEFM	UEFM change
54.7	65.7	41.7	11 L/6 R	7 f/10 m	33.3	41.4	8.03
(2.9)	(15.5)	(5.2)			(3.5)	(3.4)	(0.9)

BCI, brain–computer interface; f, female; L, left; m, male; R, right; SEM, standard error of mean; UEFM, upper extremity Fugl-Meyer assessment.

### BCI system design

**Figure 1:**
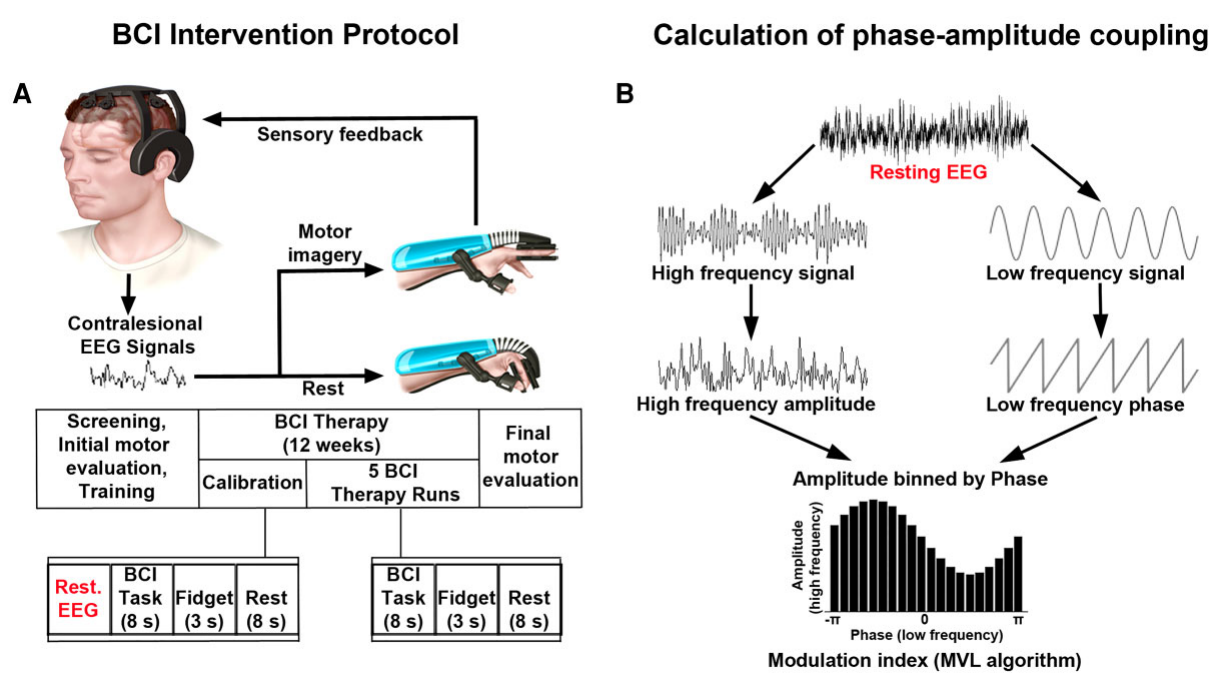
**Experimental design and EEG processing**. (**A**) BCI intervention protocol. (*Top* panels) BCI system design. Patients performed motor imagery tasks. Contralesional EEG signals were translated into commands to open or close the orthosis, which then provided proprioceptive sensory feedback to the patient as they performed motor imagery tasks. (*Bottom* panels) Intervention timeline. Patients were screened for the ability to perform the BCI task. Following screening, eligible patients underwent motor assessments before initiating the BCI therapy. Daily BCI therapy sessions included one calibration period (extended rest, alternating motor imagery and rest trials) and five BCI therapy runs (motor imagery and rest trials with active orthosis). Fidget periods were included between trials encouraging patients to blink or make physical adjustments. Motor assessments were performed every 4 weeks. Final EEG recording and motor assessment data were acquired after 12 weeks of therapy. (**B**) Data processing schematic for calculating PAC. The raw EEG signal was bandpass filtered in the lower (theta, alpha or beta) frequency range (right), and in the higher (high gamma) frequency range (left). Then, the complex analytic form of each signal was obtained using the Hilbert transform. The phase (angle of analytic signal) and power (amplitude of analytic signal) information was extracted from the lower- and higher-frequency signals, respectively. The coupling between phase and amplitude was then quantified using MVL algorithm to produce a modulation index values.

The BCI system and intervention protocol have been designed as we previously described.^23^ The system consisted of a robotic hand orthosis, EEG amplifier and wireless EEG cap with six active electrodes (US Food and Drug Administration-authorized IpsiHand Upper Extremity Rehabilitation System, Neurolutions, Santa Cruz, CA, USA) (Fig. 1A, *top* panels). A touchscreen tablet was connected via Bluetooth to the EEG headset to record signals from the brain. The local Wi-Fi network supported communication between the tablet and orthosis. The tablet guided patients through BCI tasks and translated spectral power changes into orthosis control to open and close it in a 3-finger pinch grip. For the BCI task, patients were instructed to open the orthosis with motor imagery of the affected hand or to keep the orthosis closed by resting quietly. The orthosis opened and closed in response to changes in the power of the patient-specific control signal. Subjects who could partially move their affected arm were instructed to allow passive movements by the orthotic device.

### Intervention protocol

The diagram of the BCI intervention timeline is shown in Fig. 1A (*bottom* panels). Patients were first tested for the inclusion and exclusion criteria and the ability to perform the BCI task. The exclusion criteria included severe aphasia, joint contractures in the upper limb, unilateral neglect or inability to generate a consistent BCI control signal. During EEG screening session prior to therapy implementation, patients were instructed to perform a series of rest and motor imagery trials. The 1 Hz width frequency band with spectral power modulation best corresponding to the difference between rest and motor trials was selected as the BCI device control signal. The selected control signal was always within the mu (8–12 Hz) or beta (13–29 Hz) canonical frequency band and remained consistent for each patient throughout BCI therapy. Patients with identifiable feature frequency consistent over two EEG screenings were included in the study. Patients were evaluated for baseline motor function before initiating the therapy by physical and occupational therapists. In addition to the UEFM, secondary motor function outcome measures using the AMAT, MI, MAS at the wrist and elbow and grip strength were also acquired. Research team members then trained patients in the use of the BCI system. Patients were instructed to use the device 1 h/day, 5 days/week, for a total of 12 weeks. BCI performance data per patient are provided in Supplementary Table 1. Clinicians assessed motor function once every 4 weeks. After 12 weeks of BCI therapy, patients underwent a final post-therapy motor assessment.

A session of BCI therapy took ∼1 h to complete and consisted of one calibration period and five BCI therapy runs. Pre-therapy calibration was implemented for data quality assurance and for detecting motor imagery activity during the BCI task. During calibration, patients rested quietly and then completed a series of task blocks and rest trials. During task blocks, patients were instructed to imagine moving their affected hand. The orthosis did not move during calibration. Following calibration, patients started BCI therapy runs. Each run consisted of 30 motor imagery and 30 rest trials. The trial order was randomized, and 3 s of ‘fidget’ periods were included between each 8 s trial. A ‘fidget’ periods encouraged patients to blink or make physical adjustments. After the completion of the BCI therapy run, the system paused to allow patients to rest before continuing with their therapy. Resting-state EEG data from pre-task calibration sessions were saved to a remote server for further analysis.

In order to further validate the clinical and electrophysiological effects of BCI intervention, future sham-controlled studies comparing the effects of active BCI with those observed with a sham BCI intervention are warranted. Sham BCI may consist in delivering a constant signal not coupled to the brain activity from the scalp EEG to mimic active BCI while keeping participants blind to the intervention. To rule out the possible efficacy of the sham intervention, the significance of changes in the clinical and electrophysiological outcomes between the BCI training group and the sham group should be assessed.

### EEG recording and processing

EEG was recorded by means of six wireless dry electrodes (F3, F4, C3, C4, P3 and P4) mounted on the EEG headset in an International 10–20 System (Neurolutions, Santa Cruz, CA, USA). EEG was sampled at 300 Hz with a ground electrode placed on the forehead. The electrode impedance was kept below 10 kΩ. The raw EEG data were preprocessed in a MATLAB environment (Mathworks, Natick, MA, USA). EEG data collected during the pre-therapy calibration rest period were prepared for analysis across four stages of the BCI therapy runs. These stages were Pre-BCI (before initiating the therapy), earlier Post-BCI (4th week), later Post-BCI (8th week) and final Post-BCI (12th week). Resting-state EEG data for each condition were 5 min long. For each condition, continuous EEG recording was bandpass filtered between 1 and 100 Hz using a finite impulse response (FIR) filter. To remove environmental noise, 60 Hz notch filter was applied. EEG was screened for extreme values, as well as for infrequent and unstereotyped artefacts. For further artefact attenuation, Infomax independent component analysis was applied.^74^ Independent components found to reflect eye blinks, lateral eye movements, muscle-related and cardiac artefacts were removed from the data. EEG data were common average re-referenced. Frequency bands were defined as follows: theta, 4–7 Hz; alpha, 8–12 Hz; beta, 13–29 Hz; gamma, 65–100 Hz.^38^

### Power spectral density

The power spectral density (PSD) was calculated for each condition using Welch’s method.^75^ The input signal was segmented into 50% overlapping sections each with the duration of 2 s. Each segment was windowed with a Hamming window that is the same length as the segment. A fast Fourier transform was applied to the windowed data. The periodogram of each windowed segment was averaged to form the spectrum estimate from 1 to 100 Hz. PSD values were then averaged across frequency bands and participants. The averaged data for Post-BCI runs were contrasted with Pre-BCI baseline.

A high gamma band was typically defined as cortical oscillations above 60 Hz.^76–78^ However, scalp EEG was found to effectively record high gamma activity up to 100 Hz.^48,79–83^ On the other hand, notch filtering (60 Hz) can possibly affect cortical oscillations at the neighbouring frequencies. That is why broadband gamma^84^ was defined between 65 and 00 Hz.^85^

Time–frequency analysis was additionally performed to support the idea that high gamma oscillations can be detected using scalp EEG. This analysis allowed visualizing resting high gamma cortical oscillations and their potential modulation by the BCI intervention. EEG was filtered offline using an FIR bandpass filter from 65 to 100 Hz. Data were segmented into 5 s epochs. A Morlet wavelet convolution was computed using the channel time–frequency option.^81,82^ Thirty-five linearly spaced frequencies were computed between 65 and 100 Hz. For each patient, time–frequency data were averaged across all epochs per condition. The grand average time–frequency maps were obtained by averaging data across patients (see Supplementary Fig. 1A, *top* panels). High gamma oscillations were then averaged across 65–100 Hz to visualize a single high gamma frequency wave (see Supplementary Fig. 1A, *bottom* panels).

### Phase–amplitude coupling

To calculate PAC, first, the raw signal was bandpass filtered in the frequency bands of interest (Fig. 1B). A Hilbert transform was then applied to obtain the complex-valued analytic signal. Estimates of low-frequency phase and high-frequency amplitude were extracted from the low- and high-frequency filtered analytic signal, respectively. The coupling between low-frequency phase and high-frequency amplitude was quantified using the mean vector length (MVL) approach, originally described in Canolty *et al*.^69^ PAC values were computed between phases of theta/alpha/beta frequency bands (4–7, 8–12 or 13–29 Hz) and amplitudes of the high gamma frequency band (65–100 Hz). Theta–, alpha– and beta–gamma PACs were compared between conditions. MVL approach allows us to estimate whether the power at high frequencies fluctuates systematically with the phase of the low frequency, i.e. PAC.

To rule out the possible effects of filtering on PAC results, we conducted additional analyses using neighbouring electrodes to generate the lower and higher-frequency signals to compute PAC. Neighbouring central (C3 and C4) and frontal (F3 and F4) electrodes were used for cross-electrode theta/alpha-high gamma and beta-high gamma PAC calculations, respectively.

As a complimentary tool, Canolty maps were calculated to visualize the high gamma power and theta–gamma PAC.^69^ The phase troughs of the low frequency were specified at the theta frequency band (5 Hz). A time window of 1 s was extracted around each of these troughs. A time–frequency decomposition of these short epochs was performed. The power of all the time–frequency maps was averaged to obtain the final Canolty maps (see Supplementary Fig. 1B). This approach allowed us to visualize whether the power at high frequencies fluctuated systematically with the phase of the low frequency, i.e. PAC.

### Localizing electrodes to the cortical surface for theta–gamma PAC

Cortical sources of statistically significant theta–gamma PAC increase during motor recovery relative to Pre-BCI baseline were estimated in order to spatially characterize this effect. The forward model was calculated using the Open-MEEG Boundary Element Method^86^ on the cortical surface of a template MNI brain (colin27 atlas). A noise covariance matrix was estimated from the preprocessed EEG data. Cortical source activation was calculated with a constrained inverse model of EEG sources using the weighted minimum norm current estimation^87^ and mapped to a distributed source model consisting of 15 002 elementary current dipoles. Theta–gamma PAC was computed on the source using the MVL method. We then applied voxelwise non-parametric permutation tests on PAC source space.

### Statistical analyses

Differences in the mean PAC values were examined in a repeated-measures ANOVA with within-subjects factors *Stage* (main factor with four levels: Pre-BCI, earlier, later and final Post-BCI—see the ‘EEG recording and processing’ section) × *Electrode* (F3, F4, C3, C4, P3 and P4). In case of significant interaction *Stage × Electrode* indicating an overall difference between conditions with regard to PAC as a function of the electrode, we ran separate ANOVAs for each electrode. Planned contrasts were then used to test *a priori* hypotheses and decompose the significant effects of BCI intervention. Changes in PSD values and motor assessment scores across BCI therapy runs were also assessed by repeated-measures ANOVA. All statistical tests were two-tailed with a significance level of 0.05, and the *P*-values were adjusted using a Bonferroni correction.

For the theta–gamma PAC source, under the null hypothesis of no difference between the two conditions, each point in space per subject was randomly permuted between conditions (final Post-BCI versus Pre-BCI) and the resulting data were used to compute a permutation *t*-statistic spatiotemporal map for PAC.^88–90^ Repeating this permutation procedure 1000 times, using Monte Carlo random sampling, enabled us to estimate the empirical distribution of the *t*-statistic at each voxel, and thus convert the original data into a *P*-value statistical map. Lastly, to control for multiple comparisons across all voxels, the *P*-values were adjusted using a Bonferroni correction. The significant values with *P* ≤ 0.05 were retained, while values with *P* > 0.05 were set to zero.

Correlation analyses were conducted between PAC values across BCI therapy runs to test synchrony between time series data. We used a non-parametric Spearman rank correlation to avoid imposing a model assuming a linear relation between variables.^91,92^ Correlations were also calculated between motor assessment scores and electrophysiological findings. Significance thresholds were set at *P* ≤ 0.05. It is worth noting that correlations were assessed for the statistically significant EEG effects (theta–gamma PAC increase at the C3 and C4 electrodes; beta–gamma PAC decrease at the F3 and F4 electrodes following BCI treatment). PAC values were averaged for electrodes showing significant effects, creating one value per electrode, subject and condition. The differences in PAC and motor assessment scores relative to the Pre-BCI baseline were computed, and correlation coefficients were calculated by comparing PAC and motor score changes across four stages of the BCI therapy runs.

### Data availability

The data will be made available upon reasonable request to the corresponding author.

## Results

### Motor rehabilitation

Following 12 weeks of contralesionally controlled BCI therapy, all chronic stroke patients showed an increase in UEFM score which served as a primary motor outcome assessment tool. Patients achieved a mean increase of 8.03 points in UEFM (Table 1). This increase implies clinically meaningful motor recovery surpassing the minimal clinically significant difference (MCID) threshold of 5.25 points score increase.^93^ Overall, 14 out of the 17 patients reached the MCID.

**Figure 2:**
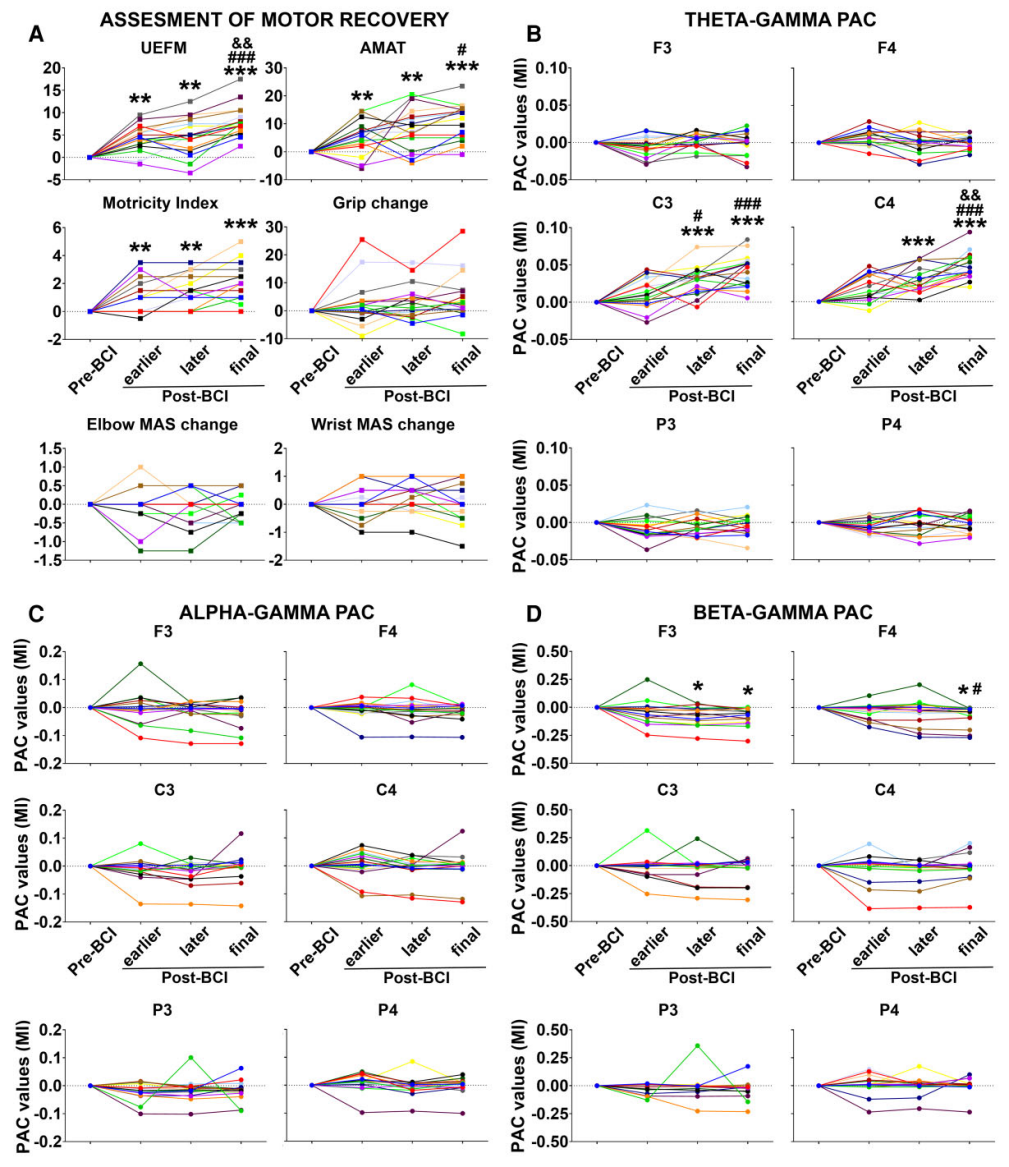
**Mean motor assessment scores and PAC values**. (**A**) Longitudinal changes in motor assessment scores from baseline through 12 weeks of BCI intervention. Each motor assessment tool represented as a separate graph. *Y*-axis, motor score; *X*-axis, stages of BCI therapy runs. UEFM, upper extremity Fugl-Meyer; AMAT, Arm Motor Ability Test; MAS, modified Ashworth Scale. (**B–D**) Theta–, alpha– and beta–gamma PAC values, respectively, across BCI therapy runs. PAC values (mean ± SEM) for each electrode from baseline through 12 weeks of BCI intervention. *Y*-axis, PAC value; *X*-axis, stages of BCI therapy runs. Patients were depicted in 17 different colours. Significance levels were based on the pairwise comparisons in ANOVA (*N* = 17; Bonferroni corrected). *, ** and *** symbols: *P* ≤ 0.05, 0.01 and 0.001 for Pre-BCI versus earlier, later or final Post-BCI contrasts; # and ### symbols: *P* ≤ 0.05 and 0.001 for earlier versus later or earlier versus final Post-BCI contrasts; && symbol: *P* ≤ 0.01 for later versus final Post-BCI contrasts; MI, modulation index; Pre-BCI: before initiating therapy; earlier Post-BCI, 4th week; later Post-BCI, 8th week; final Post-BCI, 12th week.

Figure 2A shows the mean primary and secondary motor assessment scores across four stages of the BCI therapy runs (Pre-BCI, earlier, later and final Post-BCI). Motor scores were examined in an ANOVA with within-subjects factor *Stage* (1)–(4) (see the ‘Statistical analyses’ section).

*UEFM:* the main effect of the stage proved significant, *F*(3,48) = 38.11, *P* < 0.001, indicating that UEFM scores changed across stages of BCI therapy runs. The first Helmert contrast compared motor scores during Pre-BCI with those during Post-BCI runs, revealing a significant difference for each contrast (Pre-BCI versus earlier, later or final Post-BCI, *P* = 0.002, 0.009 and 0.000003, respectively). The second and third Helmert contrasts compared Post-BCI runs with one another: earlier versus later or final Post-BCI, and later versus final Post-BCI, respectively, revealing significant differences in contrasts with final Post-BCI (earlier versus final Post-BCI and later versus final Post-BCI, *P* = 0.0008 and 0.002, respectively).

*AMAT:* the main effect of the stage proved significant, *F*(3,48) = 16.15, *P* < 0.001, indicating that AMAT scores changed across stages of BCI therapy runs. The first Helmert contrast revealed a significant difference between Pre-BCI versus Post-BCI runs (Pre-BCI versus earlier, later or final Post-BCI, *P* = 0.01, 0.002 and 0.0001, respectively). The second Helmert contrast revealed a significant difference between earlier versus final Post-BCI (*P* = 0.03). The third Helmert contrast did not yield significant results (later versus final Post-BCI, *P* = 0.08).

*Motricity index:* the main effect of the stage proved significant, *F*(3,48) = 18.71, *P* < 0.001, indicating that MI scores changed across stages of BCI therapy runs. The first Helmert contrast revealed a significant difference between Pre-BCI versus Post-BCI runs (Pre-BCI versus earlier, later or final Post-BCI, *P* = 0.007, 0.002 and 0.0001, respectively). The second and third Helmert contrasts were not significant (earlier versus later or final Post-BCI, *P* = 0.94 and 0.14, respectively; later versus final Post-BCI, *P* = 0.08).

*Grip, elbow MAS, wrist MAS:* the main effect of the stage did not prove significant, *F*(3,48) = 2.19, 0.19 and 0.87, *P* = 0.12, 0.90 and 0.46, respectively, excluding significantly changes in these motor assessment scores with the use of a BCI therapy.

### EEG effects

#### Modulation of PAC

##### Theta–gamma PAC

**Figure 3:**
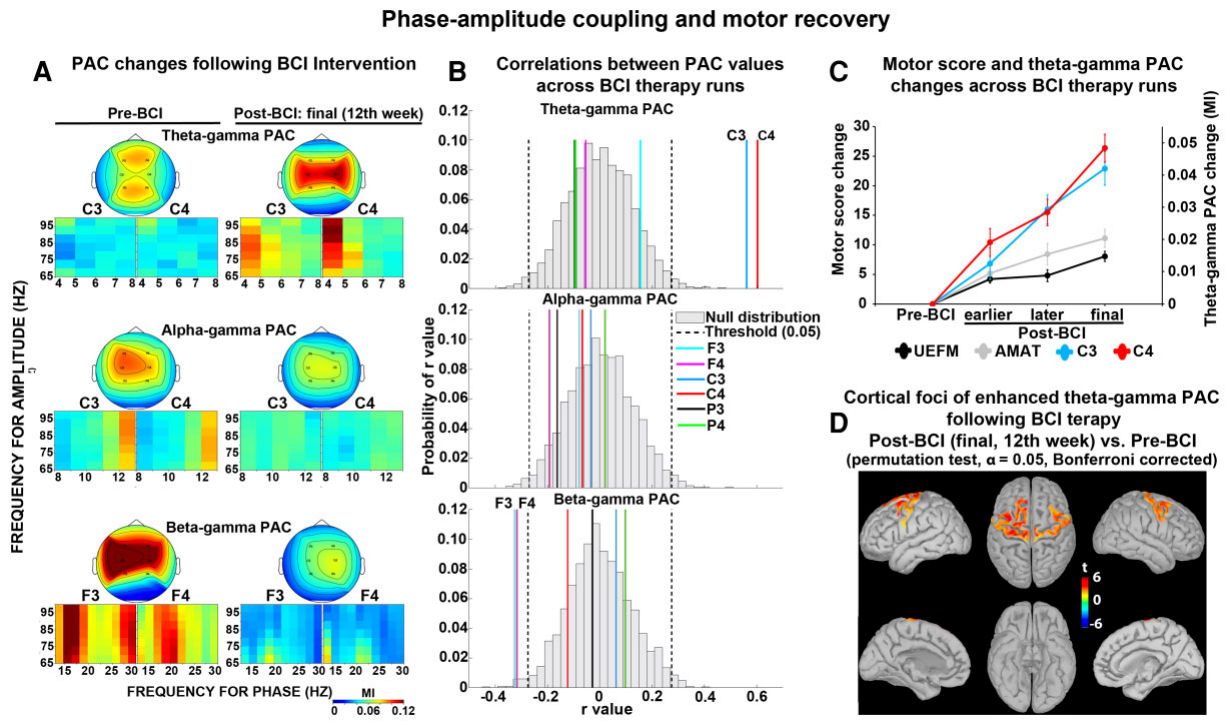
**PAC and motor recovery**. (**A**) PAC changes following BCI intervention. Coupling between the phase of theta, alpha or beta oscillations and the amplitude of gamma oscillations. (*Top* panels) Topographic distribution of PACs. (*Bottom* panels) PACs at the electrode level. *Y*-axis, frequency for amplitude (gamma range); *X*-axis, frequency for phase (theta, alpha or beta range); MI, modulation index. (**B**) Spearman rank correlations were run to calculate correlations between PAC values across BCI therapy runs (*N* = 68). Significance thresholds were set at *P* ≤ 0.05. Null distributions of Spearman rank correlation coefficients across all electrodes. *Y*-axis, probability of *r*-values; *X*-axis, *r*-value. (**C**) Longitudinal changes in motor assessment scores (UEFM and AMAT) and theta–gamma PAC values (C3 and C4 electrodes) from baseline through 12 weeks of BCI intervention. Data were shown as mean ± SEM. MI, modulation index; *Y*-axis, motor score change (left) and theta–gamma PAC change (right); *X*-axis, stages of BCI therapy runs (Pre-BCI, before initiating therapy; earlier Post-BCI, 4th week; later Post-BCI, 8th week; final Post-BCI, 12th week). UEFM, upper extremity Fugl-Meyer; AMAT: Arm Motor Ability Test. (**D**) Localizing electrodes to the cortical surface for theta–gamma PAC which correlated significantly with motor recovery. Source estimation was represented as *t*-values, based on a voxelwise non-parametric permutation tests on PAC source space. Only voxels whose *t*-statistic exceeded a critical threshold of P ≤ 0.05 (two-tailed, Bonferroni corrected) were retained. For the voxels not showing significant effects, *t*-values were set to zero.

**Figure 4:**
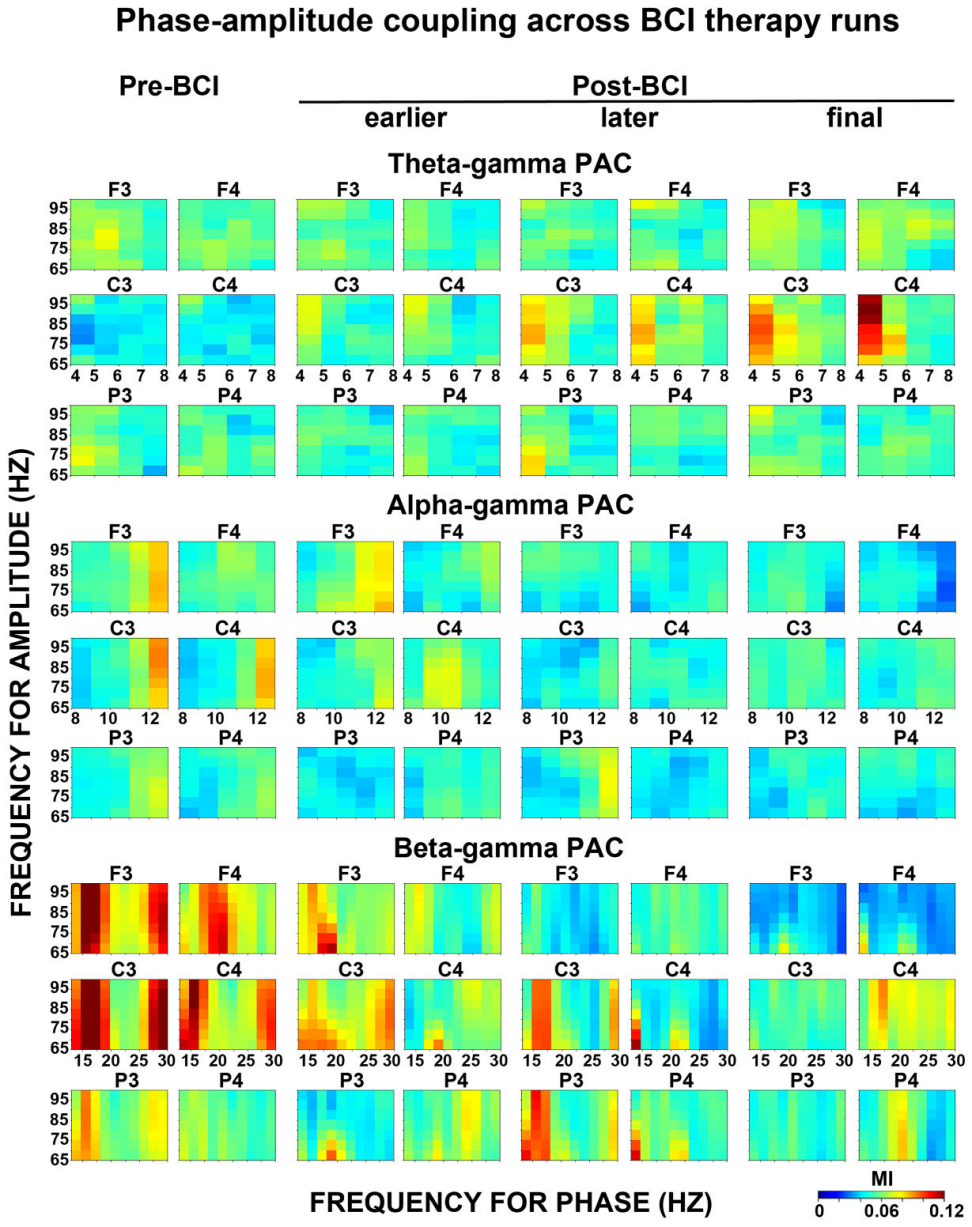
**PAC across BCI therapy runs**. Modulation of the amplitude of gamma oscillations by phase of theta, alpha or beta oscillations (*top* panels: theta–gamma PAC; *middle* panels: alpha–gamma PAC; *bottom* panels: theta–gamma PAC). PAC plots were shown for each electrode. Pre-BCI, before initiating therapy; earlier Post-BCI, 4th week; later Post-BCI, 8th week; final Post-BCI, 12th week. *Y*-axis, frequency for amplitude (gamma range); *X*-axis, frequency for phase (theta, alpha or beta range); MI, modulation index.

Figure 2B shows the mean theta–gamma PAC values across four stages of the BCI therapy runs (Pre-BCI, earlier, later and final Post-BCI) separately for each electrode (also see Figs 3A and 4, *top* panels). PACs were examined in an ANOVA with within-subjects factors *Stage* (1)–(4) × *Electrode* (1)–(6) (see the ‘Statistical analyses’ section). The main effect of the stage, *F*(3,240) = 16.48, *P* < 0.001, and interaction *Stage × Electrode*, *F*(3,240) = 17.61, *P* < 0.001, were significant, indicating an overall difference between stages with regard to PACs as a function of the electrode. We conducted separate ANOVAs for each electrode.

*C3 electrode:* the main effect of the stage proved significant, *F*(3,48) = 27.23, *P* < 0.001, indicating that PAC values changed across stages of BCI therapy runs. The first Helmert contrast revealed a significant difference between Pre-BCI versus later or final Post-BCI (*P* = 0.0004 and 0.00002, respectively), while Pre-BCI versus earlier Post-BCI contrast was not significant (*P* = 0.13). The second Helmert contrast revealed a significant difference between earlier versus later or final Post-BCI (*P* = 0.02 and *P* = 0.001, respectively). The third Helmert contrast did not yield significant results (later versus final Post-BCI, *P* = 0.16).

*C4 electrode:* the main effect of the stage proved significant, *F*(3,48) = 35.44, *P* < 0.001, indicating that PAC values changed across stages of BCI therapy runs. The first Helmert contrast revealed a significant difference between Pre-BCI versus later or final Post-BCI (*P* = 0.0002 and 0.000003, respectively), while Pre-BCI versus earlier Post-BCI contrast was not significant (*P* = 0.09). The second and third Helmert contrasts revealed a significant difference between earlier or later versus final Post-BCI (*P* = 0.001 and 0.002, respectively), while the earlier versus later Post-BCI comparison did not prove significant (*P* = 0.28).

These effects have largely been replicated when electrodes over the motor region were grouped together based on lesion side (see Supplementary Fig. 2A, left panels) indicating enhancement of theta–gamma PAC over both ipsilesional and contralesional motor cortices following BCI intervention.

*F3, F4, P3 and P4 electrodes:* the main effect of the stage did not prove significant, *F*(3,48) = 2.47, 2.36, 3.01 and 0.69, and *P* = 0.07, 0.08, 0.06 and 0.57, respectively, indicating that PACs at these electrodes were not significantly modulated with the use of a BCI therapy.

##### Alpha–gamma PAC

The same confirmatory ANOVA (see theta–gamma PAC results) was applied to examine possible alpha–gamma PAC modulation across BCI therapy runs (Fig. 2C, also see Figs 3A and 4, *middle* panels). In the *Stage* × *Electrode* ANOVA, the main effect of the stage, *F*(3,240) = 1.34, *P* = 0.22, and interaction *Stage* × *Electrode, F*(3,240) = 1.06, *P* = 0.39, did not prove significant. These findings indicate that BCI therapy did not have significant effects on alpha–gamma PAC at any electrode. The lack of alpha–gamma PAC effects was not dependent on the lesion side (see Supplementary Fig. 2A, *middle* panels).

##### Beta–gamma PAC

In the same confirmatory ANOVA (see theta–gamma PAC results), the main effect of the stage, *F*(3,240) = 0.59, *P* = 0.62, was not significant but interaction *Stage × Electrode*, *F*(3,240) = 2.13, *P* = 0.04, proved significant, indicating an overall difference between stages with regard to PACs as a function of electrode (Fig. 2D, also see Figs 3A and 4, *bottom* panels). We conducted separate ANOVAs for each electrode.

*F3 electrode:* the main effect of the stage proved significant, *F*(3,48) = 5.28, *P* = 0.001, indicating that PAC values changed across stages of the BCI therapy runs. The first Helmert contrast revealed a significant difference between Pre-BCI versus later or final Post-BCI (*P* = 0.03 and 0.01, respectively), while Pre-BCI versus earlier Post-BCI contrast was not significant (*P* = 0.93). The second and third Helmert contrasts did not reveal significant differences between Post-BCI runs (earlier versus later or final Post-BCI, *P* = 0.34 and 0.41, respectively; later versus final Post-BCI, *P* = 0.92).

*F4 electrode:* the main effect of the stage proved significant, *F*(3,48) = 4.48, *P* = 0.007, indicating that PAC values changed across stages of the BCI therapy runs. The first Helmert contrast revealed a significant difference between Pre-BCI versus final Post-BCI (*P* = 0.03), while Pre-BCI versus earlier or later Post-BCI contrasts were not significant (*P* = 0.47 and 0.91, respectively). The second Helmert contrast revealed the only significant difference between earlier versus final Post-BCI (*P* = 0.04), but earlier versus later Post-BCI contrast was not significant (*P* = 0.94). The third Helmert contrast did not prove significant (later versus final Post-BCI, *P* = 0.25).

These effects have largely been replicated when electrodes over the frontal region were grouped together based on the lesion side (see Supplementary Fig. 2A, right panels) indicating reduction of beta–gamma PAC over both ipsilesional and contralesional frontal cortices following BCI intervention.

*C3, C4, P3 and P4 electrodes:* the main effect of the stage did not prove significant, *F*(1,38) = 0.69, 1.45, 0.64 and 0.03, and *P* = 0.56, 0.24, 0.59 and 0.81, respectively, indicating that PACs at these electrodes were not significantly modulated with the use of a BCI therapy.

### Cross-electrode theta/alpha/ beta–gamma PAC

The same PAC findings have been replicated when neighbouring electrodes were used to generate the lower (theta, alpha and beta) and higher (high gamma) frequency signals to compute PAC (see Supplementary Fig. 3A and B). This excludes the possible effects of filtering on the PAC results reported above.

### Spearman correlation analyses

#### Correlations between PAC values across BCI therapy runs

Correlations between PAC values across BCI therapy runs are shown in Fig. 3B. Across-therapy run correlation coefficients for theta–gamma PAC at the C3 and C4 electrodes were 0.56 (*P* = 0.00008) and 0.60 (*P* = 0.00004), respectively, suggesting significant positive correlations. Theta–gamma PAC at the F3, F4, P3 and P4 electrodes showed poor correlations (*r* = 0.15, −0.05, −0.10, −0.09, and *P* = 0.20, 0.65, 0.42, 0.43, respectively) (Fig. 3B, *top* row). Alpha–gamma PAC did not correlate significantly across therapy runs (F3, F4, C3, C4, P3 and P4 electrodes: *r* = −0.08, −0.19, −0.03, −0.07, −0.16, 0.02, and *P* = 0.51, 0.11, 0.77, 0.58, 0.18, 0.86, respectively) (Fig. 3B, *middle* row). Beta–gamma PAC showed significant correlations at the F3 and F4 electrodes, with across-therapy run correlation coefficients of −0.34 (*P* = 0.01) and −0.33 (*P* = 0.02), respectively. Beta–gamma PAC at the C3, C4, P3 and P4 electrodes showed poor correlations (*r* = 0.06, −0.12, −0.03, 0.10 and *P* = 0.62, 0.32, 0.82, 0.43, respectively) (Fig. 3B, *bottom* row). These results have been replicated when electrodes over the right and left hemispheres were grouped together based on lesion side (see Supplementary Fig. 2B).

#### Correlations between motor recovery and theta–gamma PAC

**Figure 5:**
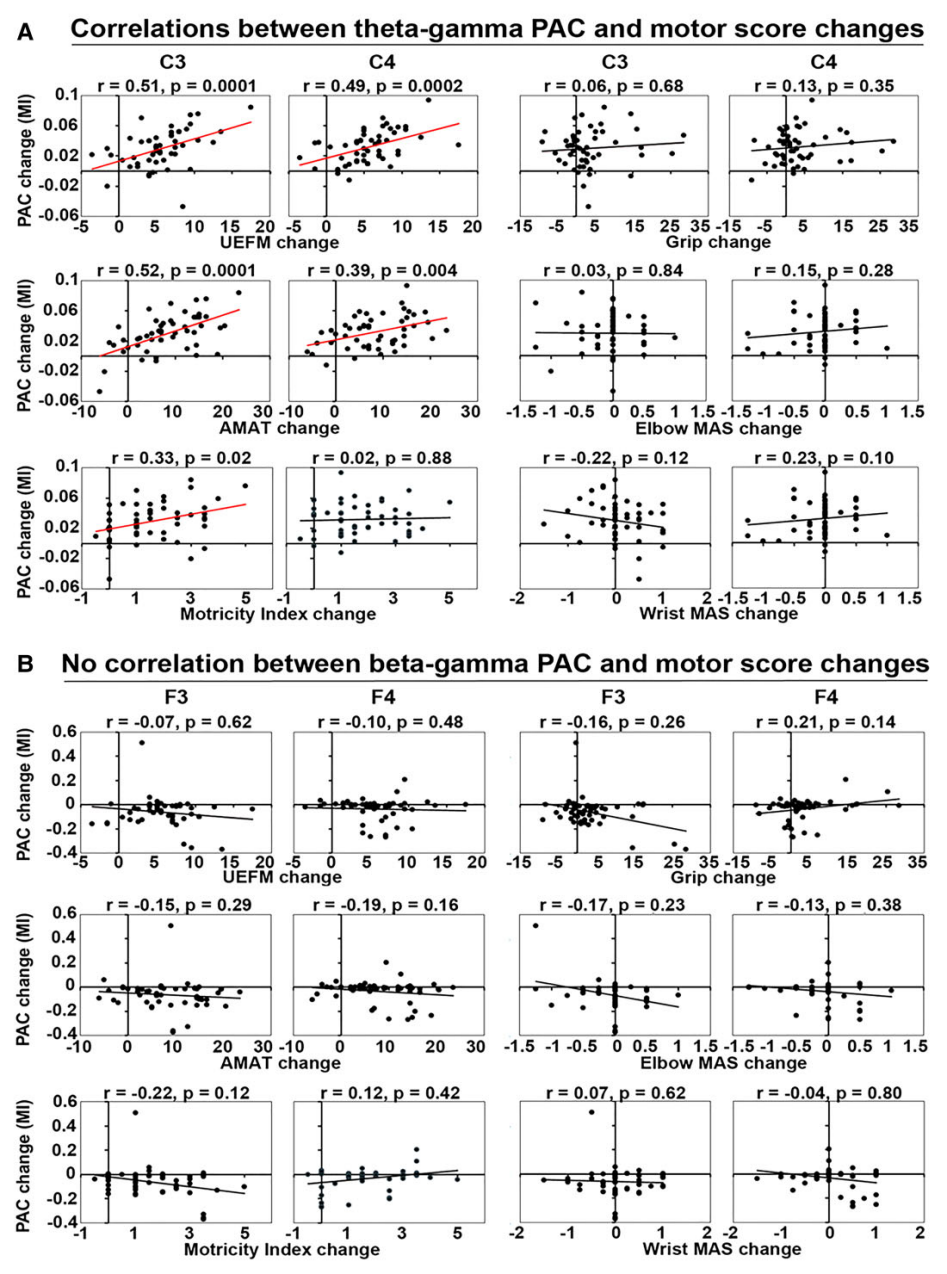
**Relationships between motor recovery and PAC change**. Spearman rank correlations were run between changes in motor scores and theta–, beta–gamma PAC values across BCI therapy runs relative to Pre-BCI baseline (*N* = 51). Significance thresholds were set at *P* ≤ 0.05. (**A**) Significant correlations between motor recovery and theta–gamma PAC changes at the C3 and C4 electrodes (UEFM: *r* = 0.51, 0.49 and *P* = 0.0001, 0.0002; AMAT: r = 0.52, 0.39 and *P* = 0.0001, 0.004; MI: *r* = 0.33 and *P* = 0.02). Other measures showed no significant correlations (MI: *r* = 0.02 and *P* = 0.88; Grip: *r* = 0.06, 0.13 and *P* = 0.68, 0.35; Elbow MAS: *r* = 0.03, 0.15 and *P* = 0.84, 0.28; Wrist MAS: *r* = −0.22, 0.23, and *P* = 0.12, 0.10). (**B**) No significant correlations have been detected between motor recovery and beta–gamma PAC changes at the F3 and F4 electrodes (UEFM: *r* = −0.07, −0.10, and *P* = 0.62, 0.48; AMAT: *r* = −0.15, −0.19, and *P* = 0.29, 0.16; MI: *r* = −0.22, 0.12 and *P* = 0.12, 0.42; Grip: *r* = −0.16, 0.21 and *P* = 0.26, 0.14; Elbow MAS: *r* = −0.17, −0.13, and *P* = 0.23, 0.38; Wrist MAS: *r* = 0.07, −0.04, and *P* = 0.62, 0.80). *Y*-axis, PAC change; *X*-axis, motor score change. MI, modulation index; UEFM, upper extremity Fugl-Meyer; AMAT: Arm Motor Ability Test; MAS: modified Ashworth Scale.

Correlations between changes in motor scores and theta–gamma PACs across BCI therapy runs relative to Pre-BCI are shown in Fig. 5A (also see Fig. 3C). Correlation coefficients between UEFM score change and PAC change at the C3 and C4 electrodes were 0.51 (*P* = 0.0001) and 0.49 (*P* = 0.0002), respectively, suggesting significant positive correlations. Similarly, AMAT score change showed significant correlation with PAC change at the C3 and C4 electrodes, with correlation coefficients of 0.52 (*P* = 0.0001) and 0.39 (*P* = 0.004), respectively. Theta–gamma PAC increase at both ipsilesional and contralesional motor electrodes showed significant correlations with UEFM and AMAT score changes (see Supplementary Fig. 2C, left panels). MI score change and PAC change at the C3 electrode correlated significantly 0.33 (*P* = 0.02), while PAC change at the C4 showed poor correlation with MI score change 0.02 (*P* = 0.88). Grip, elbow MAS and wrist MAS changes did not correlate significantly with PAC change at the C3 and C4 electrodes (*r* = 0.06, 0.13 and *P* = 0.68, 0.35; *r* = 0.03, 0.15 and *P* = 0.84, 0.28; *r* = −0.22, 0.23 and *P* = 0.12, 0.10, respectively).

#### No correlation between motor recovery and beta–gamma PAC

Correlations between changes in motor scores and beta–gamma PACs across BCI therapy runs relative to Pre-BCI are shown in Fig. 5B. UEFM, AMAT, MI, Grip, elbow MAS and wrist MAS changes correlated poorly with PAC change at the F3 and F4 electrodes (F3 electrode, *r* = −0.07, −0.15, −0.22, −0.16, −0.17, 0.07 and *P* = 0.62, 0.29, 0.12, 0.26, 0.23 and 0.62, respectively; F4 electrode, *r* = −0.10, −0.19, 0.12, 0.21, −0.13 and −0.04, and *P* = 0.48, 0.16, 0.42, 0.14, 0.38 and 0.80, respectively). The lack of correlation effects was not dependent on the lesion side (see Supplementary Fig. 2C, right panels).

### Sources of theta–gamma PAC increase following BCI therapy

To examine the sources of theta–gamma PAC increase during motor recovery relative to baseline, source estimation was calculated. Compared with Pre-BCI, the final Post-BCI resulted in significant foci of theta–gamma PAC increase (Fig. 3D). These foci were located in the cortical areas representing hand regions of the primary motor cortex on the left and right cerebral hemispheres (left-hand M1, MNI: −36, −19, 48, *P* = 0.001; right-hand M1, MNI: 38, −18, 45, *P* = 0.004).

### Power spectral density

**Figure 6:**
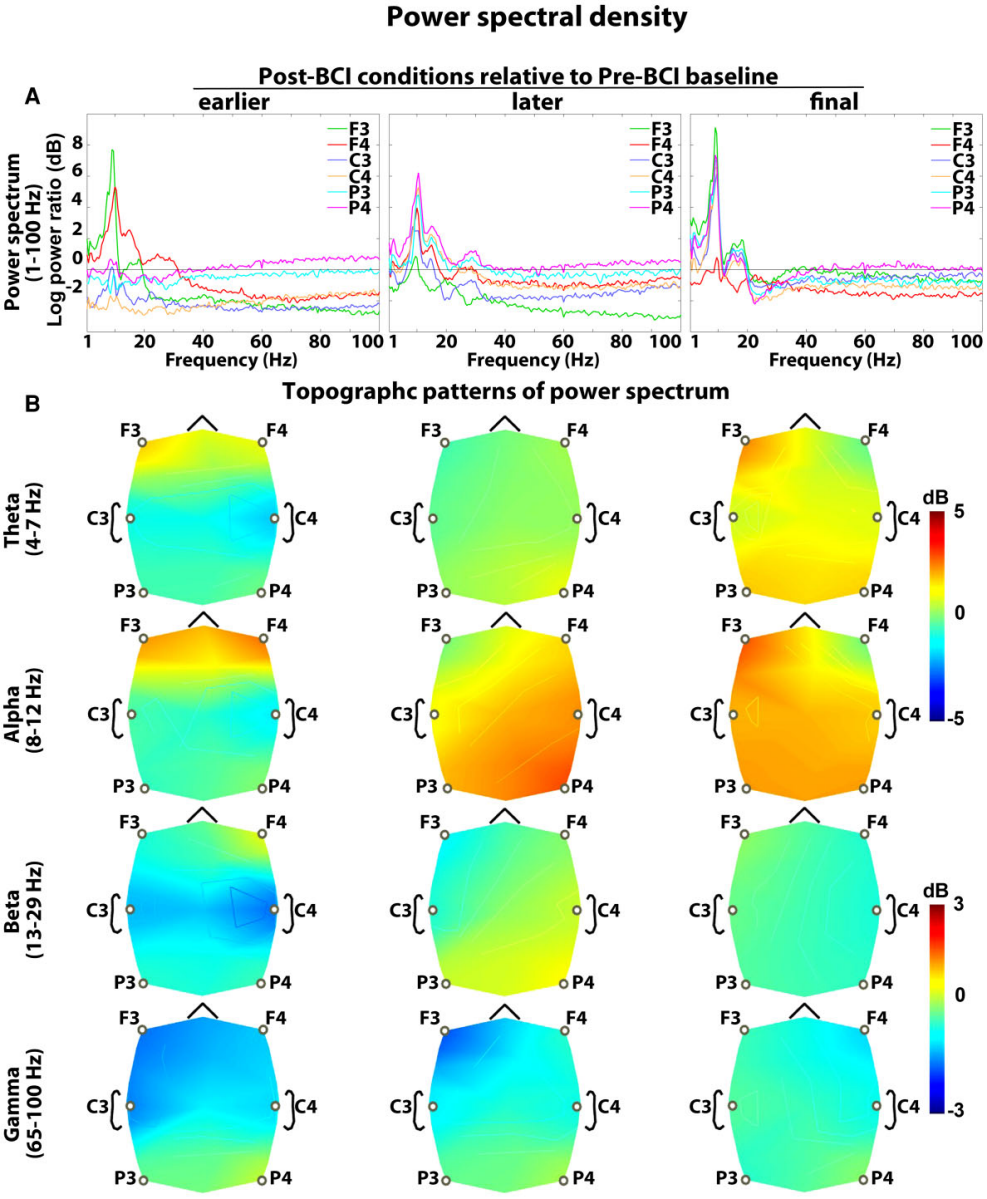
**Power spectral density**. (**A**) Average power spectra across all patients. EEG electrodes were depicted in six different colours. (**B**) Topographic representation of PSD for theta, alpha, beta and gamma frequency bands. The power spectra for Post-BCI runs were contrasted with Pre-BCI baseline. Pre-BCI, before initiating therapy; earlier Post-BCI, 4th week; later Post-BCI, 8th week; final Post-BCI, 12th week. Changes in PSD values across BCI therapy runs were assessed by repeated-measures ANOVA (*N* = 17; Bonferroni corrected). BCI intervention did not result in significant modulation of power spectrum in any frequency band.

Figure 6 shows PSD plots for Post-BCI conditions relative to Pre-BCI baseline. The same confirmatory ANOVA (see theta–gamma PAC results) was applied to each frequency band examining the possible PSD modulation with the use of a BCI therapy. In the *Stage* × *Electrode* ANOVA, the main effect of the stage did not prove significant, *F*(3,240) = 1.35, 1.22, 1.03 and 0.85, and *P* = 0.21, 0.29, 0.43 and 0.56, for theta, alpha, beta and gamma band PSDs, respectively. Likewise, the interaction *Stage* × *Electrode* was not significant, *F*(3,240) = 0.73, 1.07, 1.13 and 0.95, and *P* = 0.66, 0.41, 0.38 and 0.45, for theta, alpha, beta and gamma band PSDs, respectively. These findings indicate that BCI therapy did not have significant effects on PSDs across any frequency band or electrode, and found PAC modulation effects were not driven by underlying PSD changes.

## Discussion

In the setting of chronic stroke, this study demonstrates that motor rehabilitation using contralesionally controlled BCI training for 12 weeks induced cortical changes reflected in resting-state PAC measures. A key electrophysiological finding is the bilateral amplification of theta–gamma PAC at the C3 and C4 motor electrodes over the course of rehabilitation. Chronic stroke patients achieved clinically significant upper extremity motor recovery despite being over 6 months post-stroke. Importantly, there were significant positive correlations between theta–gamma PAC at the C3 and C4 motor electrodes, and motor assessment scores across BCI therapy runs. The sources of theta–gamma PAC increase following BCI therapy were mostly located in the hand regions of M1 on the left and right cerebral hemispheres. We also observed a bilateral decrease in beta–gamma PAC at the F3 and F4 frontal electrodes following the therapy. However, these effects did not show significant correlations with motor recovery. Moreover, alpha–gamma PAC was not modulated by BCI. Taken together, these findings support the notion that theta–gamma PAC amplification over the motor cortex is associated with functional motor improvement, and this may represent a mechanism for motor learning with the use of a BCI in chronic stroke patients.

CFC, interaction between neuronal oscillations at different frequency bands, has been gaining growing interest in the recent years.^45,55,69,94,95^ It has been described in animals,^46,47,57,96,97^ and humans,^55,69,95^ and in multiple brain regions, including hippocampus,^46,50,66^ subcortical nuclei^45,95^ and neocortex.^55,69^ Although the exact functional significance of CFC remains unclear, it has been found to manifest in response to sensory inputs and cognitive or motor tasks, and it is believed to be a major mechanism of information processing by which brain areas spatially and temporally coordinate their activity.^44,51,98^ The best-known example of CFC, namely the theta–gamma PAC, has consistently been demonstrated in relation to learning in the rodent hippocampus,^46,65–67,99^ linking this phenomenon to hippocampal function in learning and memory.^100–102^ The magnitude of theta–gamma coupling during learning of item–context associations was correlated with the high accuracy of behavioural performance, which increased during the course of learning.^54^ Studies adopting short- and working-memory paradigms have shown that theta–gamma coupling is associated with encoding and retrieval of verbal and visual information.^103,104^ These findings support the view that the theta–gamma interaction contributes to memory and learning processes. However, very little is known about the role of theta–gamma PAC in non-hippocampal-dependent learning (e.g. motor learning). It has been suggested that M1 gamma activity has pro-kinetic role that is further supported by its increase within M1 in the hyperkinetic states experienced by patients with Parkinson’s disease.^105^ Physiologically, M1 gamma activity is locked to the peaks of ongoing theta activity and thus simultaneous theta and gamma oscillatory activities in M1 show PAC.^69^ A decrease in M1 gamma-aminobutyric acid-ergic (GABAergic) activity predicts motor learning ability^106^ and represents a central mechanism for motor plasticity.^107–111^ Interestingly, theta–gamma coupling within M1 emerged spontaneously when GABA activity is blocked.^112^ Given the role of decreased GABAergic activity in motor learning and plasticity, and its relationship with theta–gamma coupling, it may be suggested that synchronization of gamma and theta oscillations represents an important signature of motor learning.

In this study, we tested a hypothesis about the role of theta–gamma PAC in motor learning. The current study is the first to characterize the dynamic changes in EEG oscillatory synchronization associated with the improvement of motor skills throughout BCI training. Our main novel finding is that the motor recovery was associated with enhanced gamma–theta coupling in the motor areas. Enhancement of theta–gamma coupling throughout BCI therapy, and most importantly, its positive correlation with motor recovery indices suggests that theta–gamma coupling is involved in the processing of motor learning. This conclusion is in agreement with the role of theta power and theta–gamma interaction in spatial and motor learning.^70,113,114^ We also found that theta–gamma PAC synchronously and constantly increased in the later therapy sessions compared with the early ones. This might reflect mechanisms promoting the development of new and more efficient motor plans and the integration of this information into a new internal model. Our findings support previous studies showing learning-related involvement of the primary sensory-motor cortex.^114–117^

In order to demonstrate the exclusive role of theta–gamma coupling in motor learning, we tested couplings between other frequency bands as well. Theta– and beta–gamma PAC both enhanced significantly following the treatment, yet only theta–gamma coupling amplification showed a significant correlation with motor recovery. Moreover, no significant effects were found with regard to alpha–gamma PAC. The lack of significant correlation of alpha– and beta–gamma PAC modulation with motor recovery emphasizes their important distinction from theta–gamma PAC in the context of BCI-driven motor recovery. It is important to note, however, that alpha– and beta–gamma PAC modulation have been associated with other motor and non-motor phenomenon. Exaggerated coupling between beta and gamma oscillations has been detected in basal ganglia, as well as motor and frontal cortices of patients with Parkinson’s disease.^118,119^ Relationship of beta–gamma PAC with motor symptoms of Parkinson’s disease is not fully understood. Nevertheless, reductions in the beta–gamma PAC through deep brain stimulation correlated with symptom improvement in Parkinson’s disease,^118,120–122^ suggesting that enhanced beta–gamma coupling might be implicated in bradykinesia and rigidity. In our study, chronic stroke patients showed enhanced beta–gamma PAC over the frontal areas which was reduced significantly following BCI intervention. The lack of correlation between bifrontal beta–gamma PAC decrease and motor recovery can be explained by the fact that the frontal cortex is predominantly involved in executive and other cognitive functions rather than motor functions.^123,124^ Thus, reduced beta–gamma coupling in frontal areas may be involved in the mechanism underlying behavioural domains outside of motor control.

In healthy humans, brief periods of low-frequency oscillations (LFOs) below 4 Hz appear at motor cortices prior to movement onset.^125,126^ Recent work has shown the role of transient movement-related LFOs in the delta and lower theta band over the motor cortical areas during skilled upper-limb tasks.^125,127–129^ Cortical circuit dysfunction after stroke led to substantially diminished LFOs in proportion to the motor deficit. The re-emergence of LFOs paralleled motor recovery, with a stronger increase in patients who showed a better recovery.^130,131^ Thus, LFOs were identified as an important neurophysiological marker of skilled motor control. In this study, we explored possible changes in resting cortical oscillatory activity following BCI intervention. It was important to establish whether coupling between neuronal oscillations at two different frequency bands was more functionally important than either of those underlying rhythms alone. We found that BCI produced modest non-significant changes in the resting power spectrum across different frequency bands. This implies that theta–gamma PAC amplification effects were driven by synchronization of underlying resting gamma and theta powers rather than changes in their magnitude. Our findings extend the body of previous work by linking the amplification of resting theta–gamma PAC dynamics in the motor cortex to motor recovery.

### Limitations

This study has several limitations worth noting. First, our sample size was limited to 17 participants. Therefore, further studies with a larger sample size to validate these preliminary results are warranted. Those studies should also include fMRI assessment to assess the effect of contralesionally driven BCI therapy on motor system functional organization. Secondly, the study was conducted under the assumption that motor deficits were stable in the chronic stage of stroke and thus we did not have a separate BCI control group. Indeed, motor deficits have been shown to improve poorly in the chronic stage of stroke.^6–8,132^ Moreover, sham BCI therapy in a different study of motor recovery in stroke patients failed to promote recovery comparable to BCI therapy.^19^ Taken together, we therefore attribute motor function improvement and associated electrophysiological changes found in this study primarily to BCI intervention. Carefully designed external multicentre studies are needed to validate the constructed model. Thirdly, our EEG recording system had a limited number of electrodes negatively affecting the spatial specificity of our findings. Finally, while the phenomenon of theta–gamma coupling was a strong finding in this study with the use of BCI, we cannot say at this time whether it is specific to BCI techniques or whether this is a more generalized phenomenon with other rehabilitation methods in the chronic phase of stroke.

## Conclusion

This study investigated the electrophysiological correlates of motor recovery in chronic stroke patients using a contralesionally controlled BCI therapy. Specifically, we tested whether theta–gamma PAC was associated with motor recovery. Concomitant with the BCI-induced functional improvement, we found enhanced theta–gamma PAC over motor regions correlated positively with these gains in motor function. These findings support the notion that specific CFC dynamics in the brain likely play a mechanistic role in mediating motor recovery in the chronic phase of stroke recovery. Further research into these neural correlates of stroke recovery will be required to define the specificity and generalizability of these frequency interactions to different therapy strategies.

## Supplementary Material

fcac136_Supplementary_Data

## Abbreviations

AMAT =: Arm Motor Ability Test
BCI =: brain–computer interface
CFC =: cross-frequency coupling
FIR =: finite impulse response
GABA =: gamma-aminobutyric acid
M1 =: primary motor cortex
MAS =: modified Ashworth Scale
MCID =: minimal clinically significant difference
MVL =: mean vector length
PAC =: phase–amplitude coupling
PSD =: power spectral densities
SEM =: standard error of mean
tACS =: transcranial alternating current stimulation
UEFM =: upper extremity Fugl-Meyer
