# Supplementary material for: Theta–gamma coupling as a cortical biomarker of brain–computer interface-mediated motor recovery in chronic stroke
Source: Brain Commun. 2022 May 25;4(3):fcac136. doi: 10.1093/braincomms/fcac136 (PMC9188323; doi:10.1093/braincomms/fcac136)
Supplement: fcac136_Supplementary_Data [file fcac136_supplementary_data.pdf]

Supplementary materials for:

Theta-Gamma Coupling as a Cortical Biomarker of Brain-Computer Interface Mediated Motor Recovery in Chronic Stroke

Nabi Rustamov [1, 2, *], Joseph Humphries [3], Alexandre Carter [4], Eric C. Leuthardt [1, 2, 3, 5, 6 *]

Author affiliations:

1 Department of Neurological Surgery, Washington University School of Medicine, St. Louis, MO, United States of America

2 Center for Innovation in Neuroscience and Technology, Washington University School of Medicine, St. Louis, MO, United States of America

3 Departments of Biomedical Engineering, Washington University in St. Louis, St. Louis, MO, United States of America

4 Departments of Neurology, Washington University in St. Louis, St. Louis, MO, United States of America

5 Department of Neuroscience, Washington University School of Medicine, St. Louis, MO, United States of America

6 Department of Mechanical Engineering and Materials Science, Washington University in St. Louis, St. Louis, MO, United States of America

*** Correspondence to: Eric C. Leuthardt MD, FNAI**

Email: leuthardte@wustl.edu

*** Correspondence may also be sent to: Nabi Rustamov MD, PhD**

Email: rustamov.nabi@wustl.edu

Supplementary Table 1. Brain-computer interface (BCI) performance data.

Patient	BCI usage (hours)	Trials (with at least 10% move and rest accuracy)	BCI sessions (trials/5)
1	7.7	46	9.2
2	35.2	211	42.2
3	10.7	64	12.8
4	24.2	145	29
5	30.5	183	36.6
6	42.0	252	50.4
7	41.5	249	49.8
8	55.7	334	66.8
9	70.2	421	84.2
10	40.3	242	48.4
11	53.8	323	64.6
12	24.5	147	29.4
13	80.0	480	96
14	72.2	433	86.6
15	33.8	203	40.6
16	62.2	373	74.6
17	24.3	146	29.2

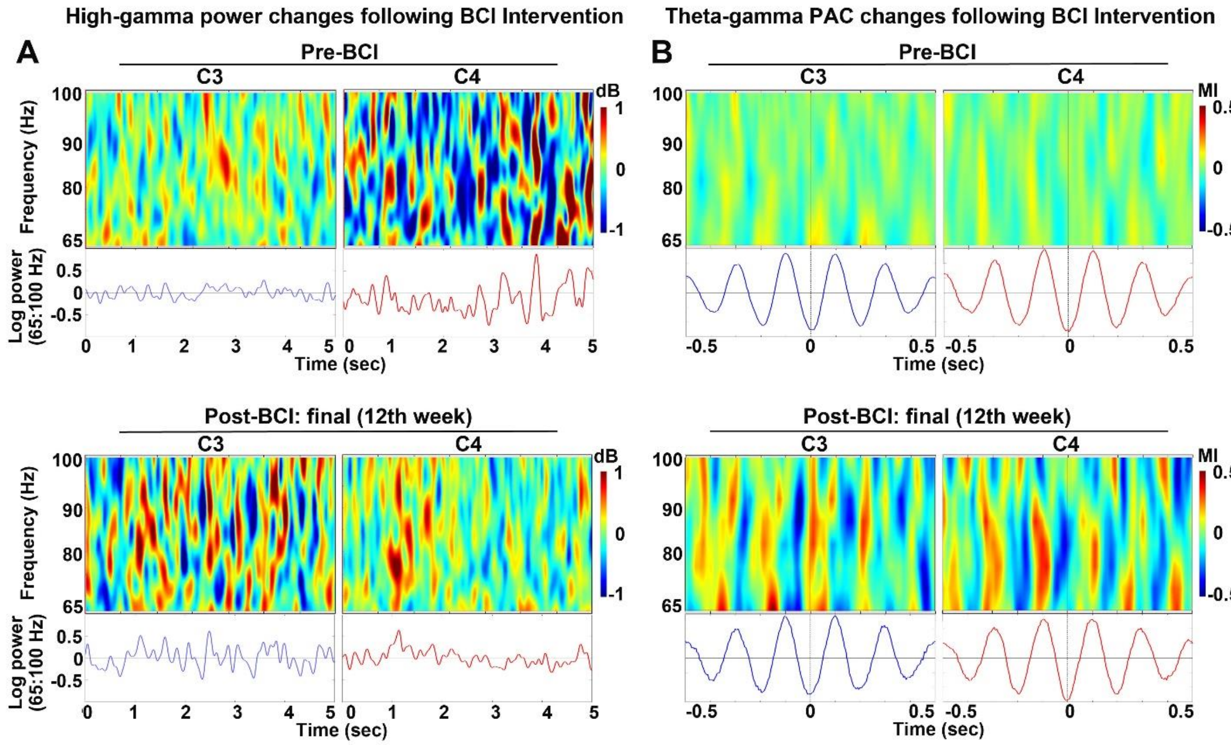

Supplementary Figure 1. High gamma power. (A) Time-frequency representation of cortical gamma oscillations following BCI intervention. Top panels: high gamma oscillations between 65 and 100 Hz. Positive and negative power fluctuations were represented by red and blue colors, respectively. X-axis, time in sec; Y-axis, frequency in Hz. Bottom panels: averaged high gamma power across 65:100 Hz. X-axis, time in sec; Y-axis, amplitude; dB: decibel. **(B)** Theta-gamma PAC changes following BCI intervention. Modulation of the amplitude of gamma oscillations by phase of theta oscillations. Top panels: amplitude of high gamma oscillations between 65 and 100 Hz. Positive and negative relationships were depicted by red and blue colors, respectively. Bottom panels: phase troughs of the low frequency specified at theta frequency band (5 Hz). MI: Modulation Index.

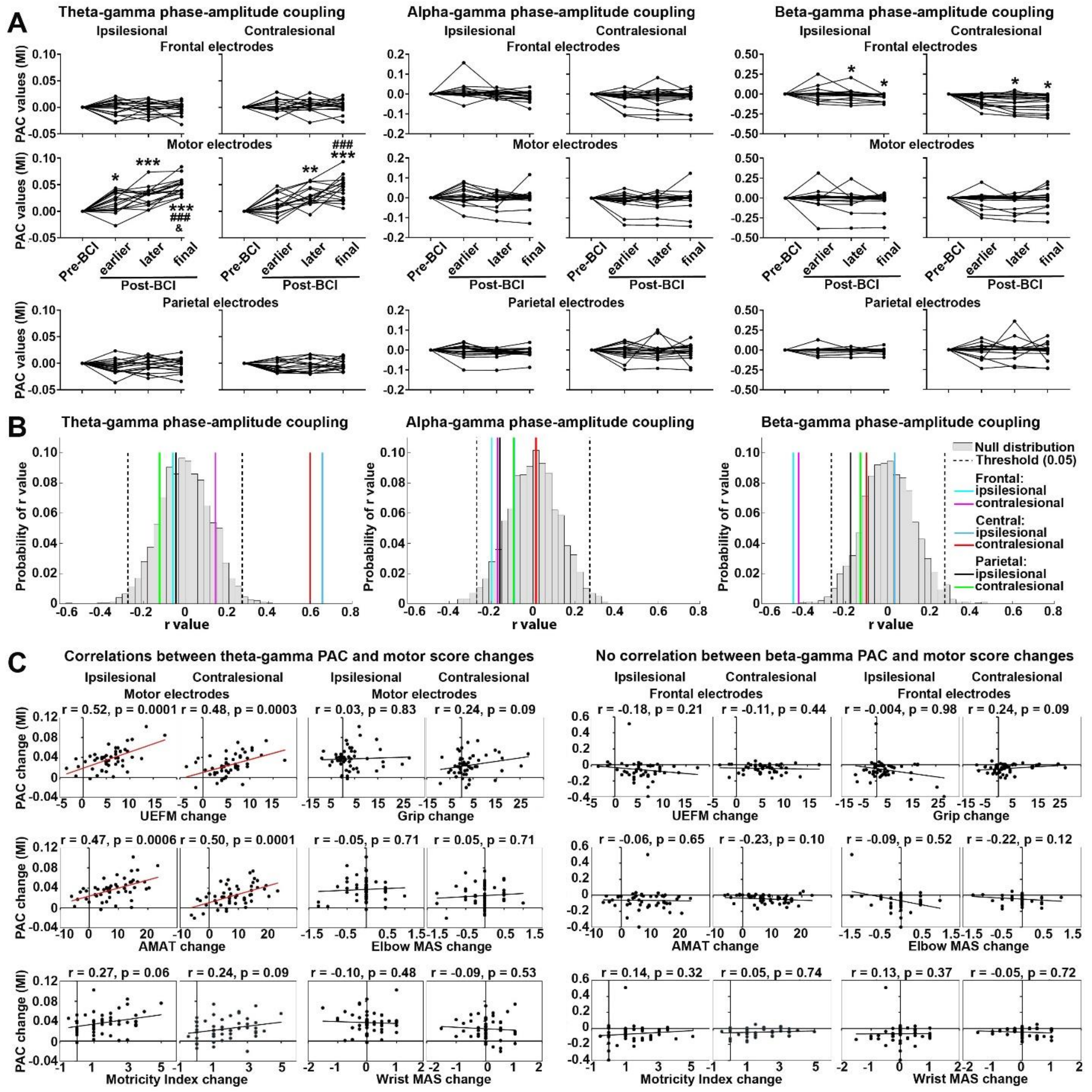

Supplementary Figure 2. Ipsilesional and contralesional changes in PAC following BCI therapy. (A) Theta-, alpha- and beta-gamma PAC values (mean ± SEM) for each electrode from baseline through 12 weeks of BCI intervention. Y-axis, PAC value; X-axis, stages of BCI therapy runs; MI: Modulation Index. Significance levels were based on the Pairwise Comparisons in ANOVA (N = 17; Bonferroni corrected). *, **, *** symbols: $p \leq 0.05$, 0.01, and 0.001 for Pre-BCI vs. earlier, later or final Post-BCI contrasts; ### symbol: 0.001 for earlier vs. later or earlier vs. final Post-BCI contrasts; & symbol: $p \leq 0.05$ for later vs. final Post-BCI contrasts. Pre-BCI: before initiating therapy; earlier Post BCI: 4th week; later Post-BCI: 8th week; final Post-BCI: 12th

week. *Theta-gamma PAC:* ipsilesional motor electrode, there were significant differences between Pre-BCI vs. earlier, later or final Post-BCI. Contrasts between earlier or later vs. final Post-BCI proved significant; contralesional motor electrode, there was a significant difference between Pre-BCI vs. later or final Post-BCI. Contrast between earlier vs. final Post-BCI proved significant. *Alpha-gamma PAC* was not altered by BCI therapy. *Beta-gamma PAC:* both ipsilesional and contralesional F3 and F4 electrodes showed significant differences between Pre-BCI vs. later or final Post-BCI. Other electrodes did not show significant effects. **(B)** Spearman rank correlations were run to calculate correlations between PAC values across BCI therapy runs (N = 68). Significance thresholds were set at $p \leq 0.05$. Null distributions of Spearman rank correlation coefficients across all electrodes. Y-axis, probability of r values; X-axis, r value. *Theta-gamma PAC* at the ipsilesional and contralesional motor electrodes correlated significantly across therapy runs ($r = 0.68$, $p = 0.000008$, and $r = 0.60$, $p = 0.00004$, respectively). *Alpha-gamma PAC* did not show significant correlations. *Beta-gamma PAC* at the ipsilesional and contralesional frontal electrodes correlated significantly across-therapy runs ($r = -0.42$, $p = 0.006$, and -0.44, $p = 0.001$, respectively). Other electrodes did not show significant correlations. **(C)** Relationships between motor recovery and PAC change. Spearman correlations were run between changes in motor scores and theta-, beta-gamma PAC values across BCI therapy runs relative to Pre-BCI baseline (N = 51). Significance thresholds were set at $p \leq 0.05$. Y-axis, PAC change; X-axis, motor score change. UEFM: Upper Extremity Fugl-Meyer; AMAT: Arm Motor Ability Test; MAS: modified Ashworth Scale. *Theta-gamma PAC:* UEFM and AMAT score changes showed significant correlations with PAC change at the ipsilesional and contralesional motor electrodes (depicted by red color). UEFM: r = 0.52, 0.48, and p = 0.0001, 0.0003; AMAT: r = 0.47, 0.50, and p = 0.0006, 0.0001. *Beta-gamma PAC:* motor recovery correlated poorly with PAC change at the ipsilesional and contralesional frontal electrodes.

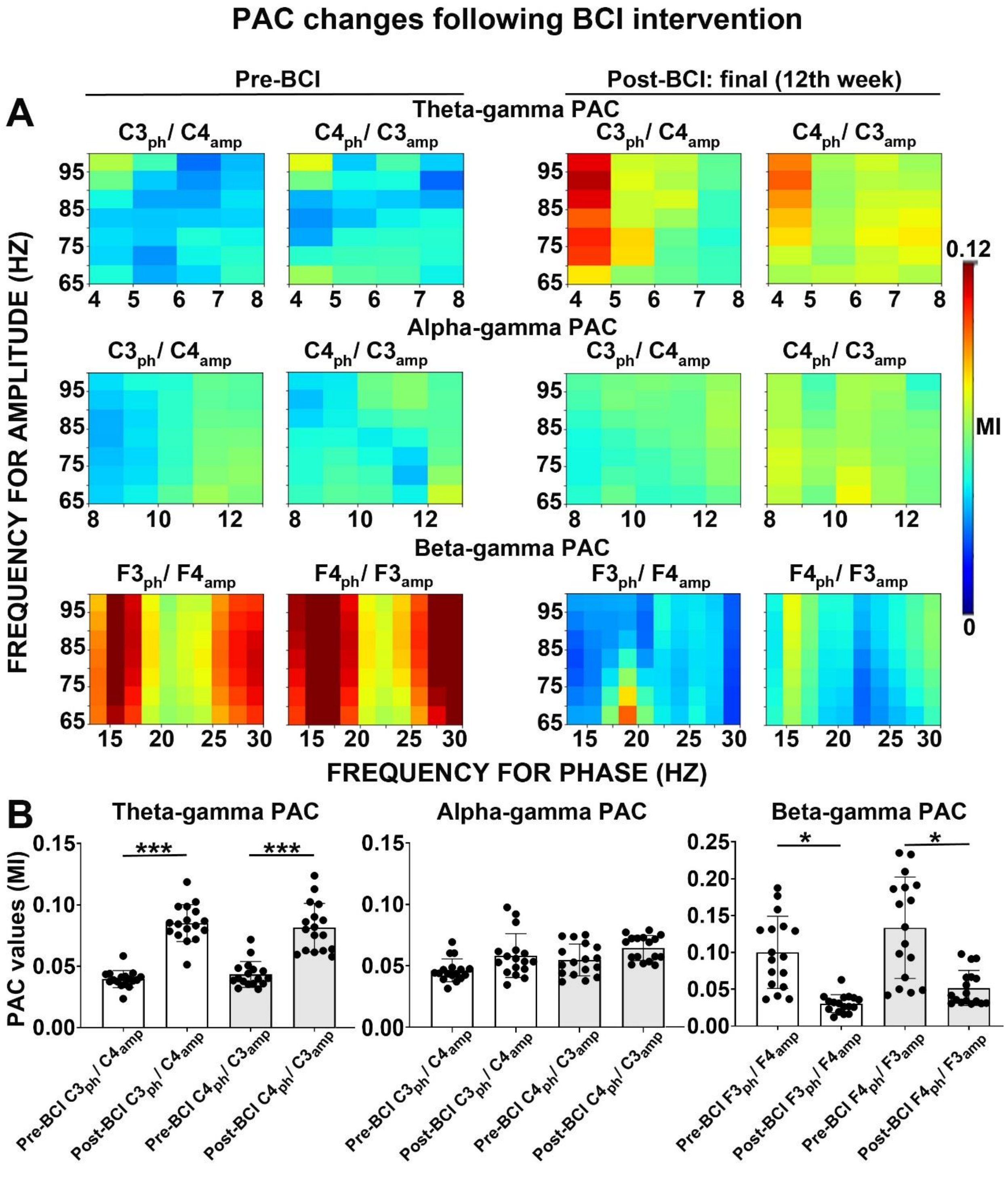

Supplementary Figure 3. PAC changes following BCI intervention. (A) Coupling between the phase of theta, alpha or beta oscillations and the amplitude of gamma oscillations. Phase of lower frequency and amplitude of higher frequency were computed from neighboring electrodes (C3 and C4 for theta/alpha-gamma PAC; F3 and F4 for beta-gamma PAC). Higher coupling values were

depicted by red color. Y-axis, frequency for amplitude (high gamma range); X-axis, frequency for phase (theta, alpha or beta range); MI: Modulation Index; ph.: phase; amp.: amplitude. **(B)** Mean values for theta-, alpha- and beta-gamma PAC. PAC values (mean ± SD) for each electrode before and 12 weeks after BCI intervention. Y-axis, PAC value; X-axis, stages of BCI intervention; MI: Modulation Index. Significance levels were based on the Pairwise Comparisons in ANOVA (N = 17; Bonferroni corrected). *** symbol: $p \leq 0.001$, * symbol: $p \leq 0.05$ for Pre-BCI vs. final Post-BCI contrasts. Pre-BCI: before initiating therapy; final Post-BCI: 12th week.
